# Cutaneous smooth muscle tumors associated with Epstein-Barr virus in an adult patient with HIV^☆^^☆☆^

**DOI:** 10.1016/j.abd.2020.06.010

**Published:** 2021-02-04

**Authors:** Estefania Galeano-Piedrahita, Ana Maria Maya Rico, Ana Cristina Ruiz Suárez, Andrea Laverde Walter

**Affiliations:** Dermatology Service, CES University, Medellín, Antioquia, Colombia

**Keywords:** Epstein-Barr virus infections, HIV, Skin manifestations, Smooth muscle tumor

## Abstract

Epstein Barr virus-associated smooth muscle tumors are an uncommon neoplasm that occurs in immunosuppressed patients of any age. Usually, it presents as multifocal tumors mainly in the spinal cord, epidural region, gastrointestinal tract and liver, upper respiratory tract and skin, the latest with few cases reported in the literature and related with human immunodeficiency virus infection and acquired immune deficiency syndrome. The authors present the first case of a Colombian adult patient with human immunodeficiency virus infection and multifocal Epstein Barr virus-associated smooth muscle tumors in the skin and epidural region, confirmed by histopathology, immunohistochemistry and in situ hybridization studies.

## Introduction

Epstein-Barr virus (EBV)-associated smooth muscle tumors are a rare neoplasm associated with immunosuppression.^1^ Their relationship with EBV has been studied extensively for more than 20 years and they have been documented in both pediatric and adult patients, with a slight female predominance.^2^, ^3^ Skin manifestations are uncommon and have been related to patients with human immunodeficiency virus (HIV) infection.^4^ The following report describes a confirmed case of a patient with HIV and presence of multifocal cutaneous and epidural smooth muscle neoplasms.

## Case report

**Figure 1:**
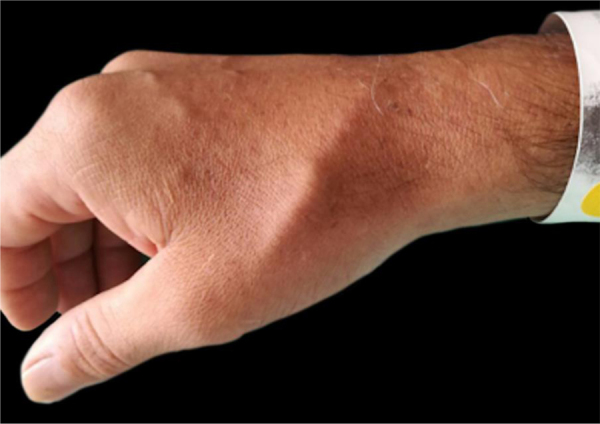
Linear subcutaneous nodule on the dorsum of the right hand.

**Figure 2:**
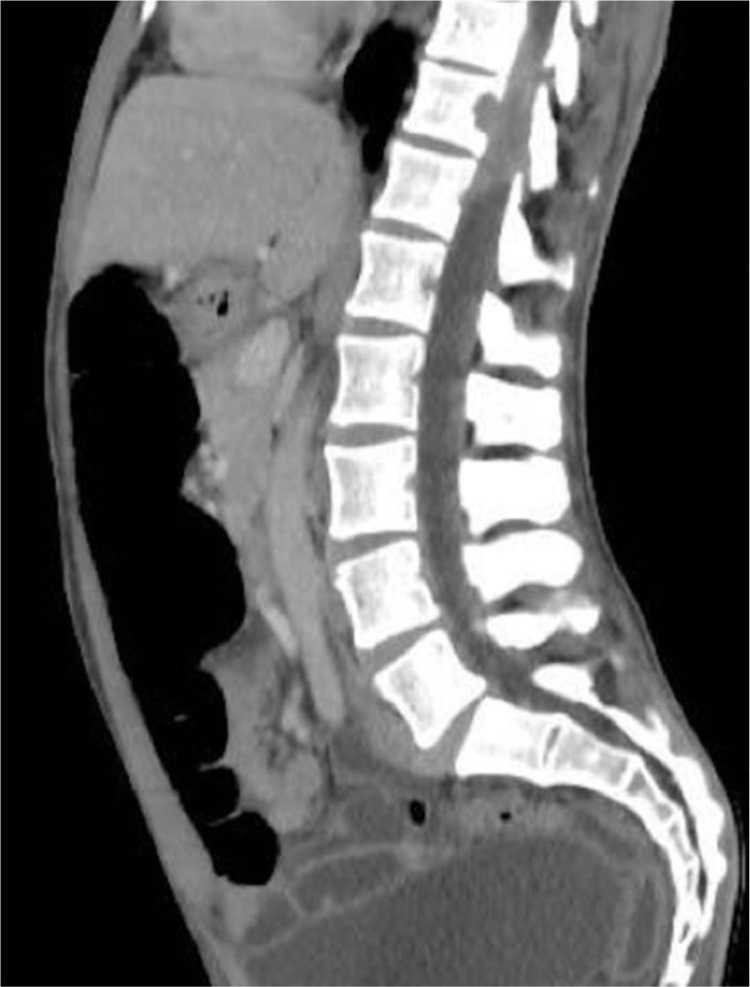
Nuclear magnetic resonance of the spine with epidural infiltration and foraminal involvement of T11 and T12, with compression and displacement of the spinal cord.

**Figure 3:**
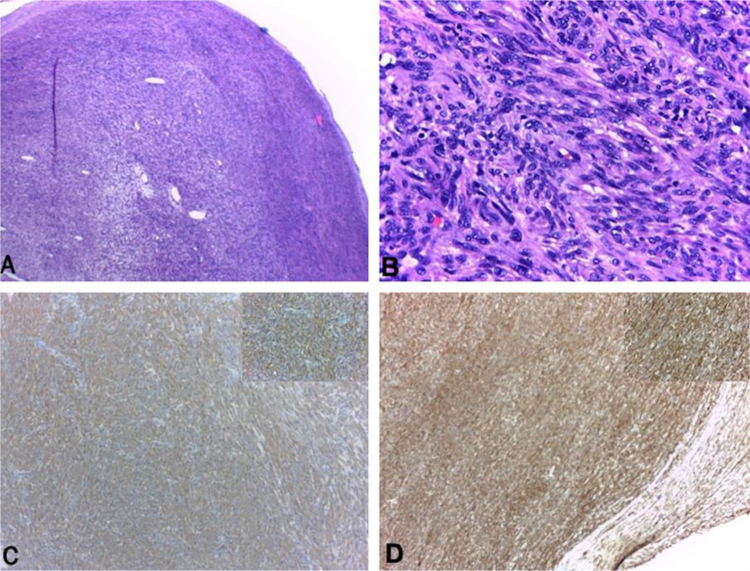
Subcutaneous nodule. (A and B), well circumscribed nodular formation formed by spindle cell bundles with minimal atypia and few mitotic figures (Hematoxylin & eosin ×100 and ×400, respectively). (C and D), SMA and H-caldesmon positive immunohistochemistry, respectively.

**Figure 4:**
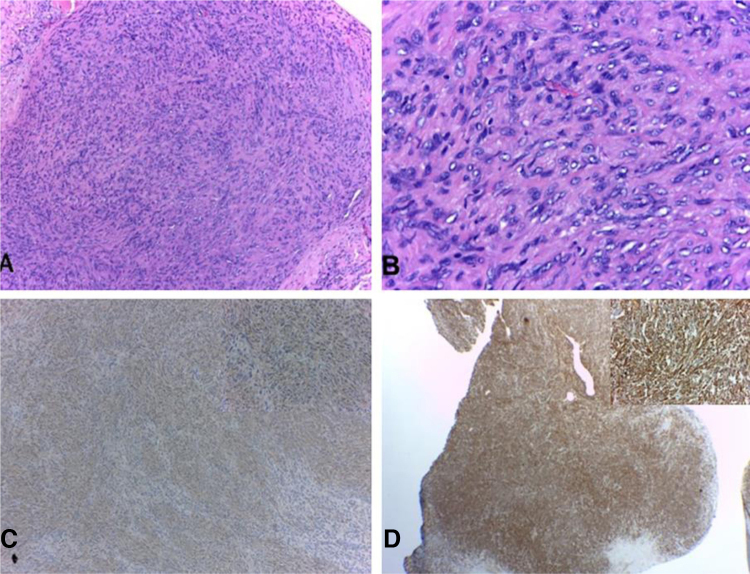
Epidural lesion. (A and B), confused lesion by spindle cell bundles with low atypia (Hematoxylin & eosin ×100 and ×400, respectively). (C and D), SMA and H-caldesmon positive immunohistochemistry, respectively.

**Figure 5:**
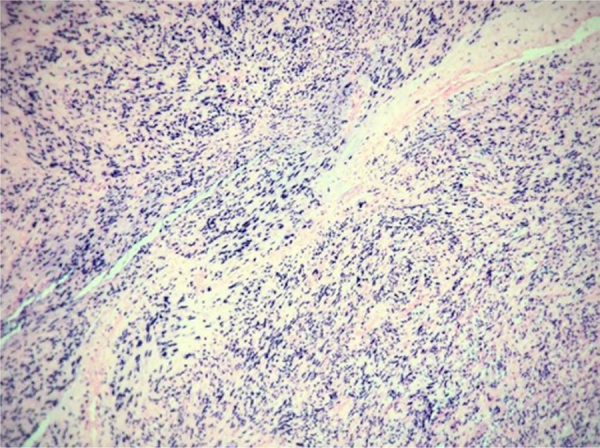
*In situ* hybridization study for detection of Epstein-Barr virus.

40-year-old male patient with a diagnosis of HIV infection since 2006 in low-adherence antiretroviral therapy, who presents a two-year history of the appearance of four painful, slow-growing lesions in the limbs and abdomen, associated with low back pain. The physical examination showed well defined subcutaneous nodules, mobile, of a rubbery consistency and very painful, located in the left forearm (1 × 1.5 cm), abdomen (0.5 × 0.5 cm), left thigh (1 × 0.5 cm), and one of larger size and linear shape on the dorsum of the right hand (2 × 1 cm) (Fig. 1). Within the relevant laboratory, 2,200 leukocytes, 920 lymphocytes, with CD4 of 6 cells/mcL and a viral load of 36,600 copies are reported. In the magnetic resonance imaging (MRI) of the contrasted spine, an extensive epidural infiltration with foraminal involvement of T4-T5, T7, T11, and T12 with compression and displacement of the spinal cord is evident (Fig. 2). A biopsy of one of the skin lesions (Fig. 3) and of the epidural mass (Fig. 4) were taken with findings similar to histology, finding a positive fusocellular neoplasm for H-caldesmon and smooth muscle actin (SMA) a diagnosis of smooth muscle tumor. Subsequently, an *in situ* hybridization study was performed for the detection of the Epstein-Barr virus encoding region (EBER), which was positive (Fig. 5).

## Discussion

The relationship between smooth muscle tumors and immunosuppression was first documented in 1970 by Pritzker et al., and it was only in1995 that their link to EBV was identified.^2^, ^5^ This association has been established in post-transplanted patients, with primary immunodeficiencies and HIV, mainly in those with CD4 less than 200 cells/mcL.^6^ The majority present multifocal compromise, as in the case of the current patient, with a predominance in the spinal cord, epidural space, gastrointestinal tract and liver, upper respiratory tract, and skin, the latter organ having few cases reported in the literature and none in Colombia.^1^, ^3^, ^4^

Although its pathogenesis is poorly understood, it has been suggested that EBV infects smooth muscle cells directly by adhering to CD21 facilitating the replication of these cells.^2^ Other theories are based on the overexpression of the proto-oncogene MYC and the activation of the AkT/m TOR pathway triggered by the LMP2A protein, which promotes cell proliferation and tumor formation.^7^, ^8^

The diagnosis is made based on physical examination, radiology, and findings in the histological and immunohistochemical study; it is confirmed with *in situ* hybridization study for the detection of the Epstein-Barr virus, as was done in the representative case.^9^ The presence of these lesions in the central nervous system has been associated with a worse prognosis; regarding treatment, there are some case reports of reversal of the neoplasms with the improvement of immunosuppression, surgical resection for isolated lesions have been published, and the use of radiotherapy and chemotherapy such as sirolimus.^3^ However, most reports are in patients without HIV infection and the results have not been completely satisfactory.^1^, ^10^

## Financial support

None declared.

## Authors’ contributions

Estefania Galeano-Piedrahita: Approval of the final version of the manuscript; study conception and planning; preparation and writing of the manuscript; data collection, analysis, and interpretation; effective participation in research orientation; intellectual participation in propaedeutic and/or therapeutic conduct of studied cases; critical review of the literature; critical review of the manuscript.

Ana Maria Maya Rico: Approval of the final version of the manuscript; study conception and planning; preparation and writing of the manuscript; data collection, analysis, and interpretation; effective participation in research orientation; intellectual participation in propaedeutic and/or therapeutic conduct of studied cases; critical review of the literature; critical review of the manuscript.

Ana Cristina Ruiz Suárez: Approval of the final version of the manuscript; study conception and planning; preparation and writing of the manuscript; data collection, analysis, and interpretation; effective participation in research orientation; intellectual participation in propaedeutic and/or therapeutic conduct of studied cases; critical review of the literature; critical review of the manuscript.

Andrea Laverde Walter: Approval of the final version of the manuscript; study conception and planning; preparation and writing of the manuscript; data collection, analysis, and interpretation; effective participation in research orientation; intellectual participation in propaedeutic and/or therapeutic conduct of studied cases; critical review of the literature; critical review of the manuscript.

## Conflicts of interest

None declared.
